# Supplementary material for: Biscuits Enriched with Monofloral Bee Pollens: Nutritional Properties, Techno-Functional Parameters, Sensory Profile, and Consumer Preference
Source: Foods. 2022 Dec 21;12(1):18. doi: 10.3390/foods12010018 (PMC9818951; doi:10.3390/foods12010018)
Supplement: Supplementary file 1 [file foods-12-00018-s001.zip › foods-2103936-supplementary.pdf]

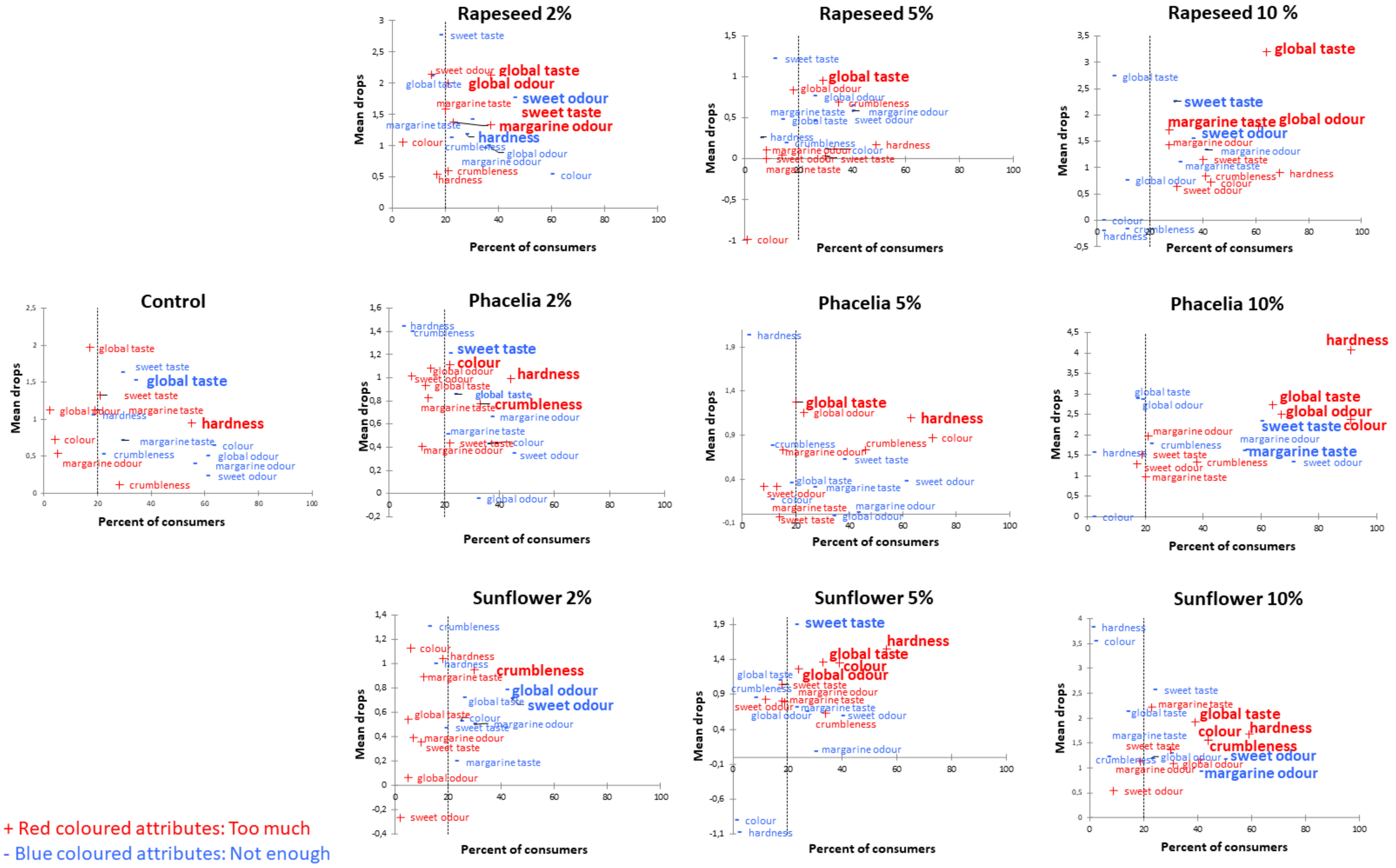

Figure S1. Penalty analysis of biscuits (Mean drop and overall penalty are significant ($p < 0.05$) for highlighted attributes)
